# Efficient G protein coupling is not required for agonist‐mediated internalization and membrane reorganization of the adenosine A_3_ receptor

**DOI:** 10.1096/fj.202001729RR

**Published:** 2021-03-12

**Authors:** Leigh A. Stoddart, Laura E. Kilpatrick, Ross Corriden, Barrie Kellam, Stephen J. Briddon, Stephen J. Hill

**Affiliations:** ^1^ Cell Signalling and Pharmacology Research Group, Division of Physiology, Pharmacology and Neuroscience, School of Life Sciences University of Nottingham Nottingham UK; ^2^ Centre of Membrane Proteins and Receptors (COMPARE) University of Birmingham and University of Nottingham Midlands UK; ^3^ School of Pharmacy Biodiscovery Institute, University Park Nottingham Nottingham UK

**Keywords:** β‐arrestin, adenosine A_3_ receptor, fluorescence correlation spectroscopy, G protein‐coupled receptor, internalization, membrane organization

## Abstract

Organization of G protein‐coupled receptors at the plasma membrane has been the focus of much recent attention. Advanced microscopy techniques have shown that these receptors can be localized to discrete microdomains and reorganization upon ligand activation is crucial in orchestrating their signaling. Here, we have compared the membrane organization and downstream signaling of a mutant (R108A, R3.50A) of the adenosine A_3_ receptor (A_3_AR) to that of the wild‐type receptor. Fluorescence Correlation Spectroscopy (FCS) studies with a fluorescent agonist (ABEA‐X‐BY630) demonstrated that both wild‐type and mutant receptors bind agonist with high affinity but in subsequent downstream signaling assays the R108A mutation abolished agonist‐mediated inhibition of cAMP production and ERK phosphorylation. In further FCS studies, both A_3_AR and A_3_AR R108A underwent similar agonist‐induced increases in receptor density and molecular brightness which were accompanied by a decrease in membrane diffusion after agonist treatment. Using bimolecular fluorescence complementation, experiments showed that the R108A mutant retained the ability to recruit β‐arrestin and these receptor/arrestin complexes displayed similar membrane diffusion and organization to that observed with wild‐type receptors. These data demonstrate that effective G protein signaling is not a prerequisite for agonist‐stimulated β‐arrestin recruitment and membrane reorganization of the A_3_AR.

**Abbreviations**
A_3_AR: adenosine A_3_ receptor
BiFC: bimolecular fluorescence complementation
CRE‐SPAP: cAMP response element‐secreted placental alkaline phosphatase
FCS: fluorescence correlation spectroscopy
FSK: forskolin
GPCR: G protein‐coupled receptor
PCH: photon counting histogram
PTx: pertussis toxin
R: inactive receptor
R*: active receptor

## 1 INTRODUCTION

The adenosine A_3_ receptor (A_3_AR) belongs to a subfamily of four G protein‐coupled receptors (GPCRs) that are all activated by their cognate ligand adenosine.^1^ The A_3_AR is expressed in a range of tissues and its activation is thought to play an important role in a variety of disease states such as rheumatoid arthritis, ischemic cardiovascular conditions, and neuropathic pain.^2^ In addition, the A_3_AR is known to be overexpressed in a variety of cancers.^3^ Therapeutics targeting this receptor are under development for the treatment of neuropathic pain, although there is still some debate regarding whether an A_3_‐agonist or antagonist would be clinically beneficial.^4^ This differing role of the A_3_AR under different pathological settings raises the possibility that targeting a specific signaling pathway may be advantageous.

GPCRs are dynamic proteins that can exist in multiple states ranging from a fully inactive conformation to a fully active conformation.^5^ In addition, it has recently been shown that there is not just one active and inactive conformation, but also multiple different active and inactive conformations can exist.^6^ The interplay between these states can be influenced by the presence of agonist or antagonist molecules that bind the receptor, intracellular binding proteins such as G proteins and arrestins, and the composition of the cell membrane environment.^6^, ^7^, ^8^ In addition, it is becoming clear that GPCRs are not uniformly distributed at the cell surface and that some GPCRs are organized within membrane compartments and microdomains.^9^, ^10^ The precise organization of receptors at the cell surface is the focus of intense study using a range of techniques and the interplay of all these elements on receptor activity allows one receptor to give rise to multiple signaling outcomes.

The recent advances in single molecule microscopy have seen a range of these techniques applied to study the heterogeneity of GPCRs at the single cell level.^9^ One such technique, fluorescence correlation spectroscopy (FCS), measures the temporal fluctuation of fluorescent species as they pass through a small defined confocal volume.^11^ By examining the changes in these fluctuations over time through autocorrelation analysis, information on the number of mobile particles in the volume (*N*) and the average dwell time of the molecules (*τ_D_*) can be obtained. As the measurement volume can be quantitatively defined, *τ_D_* can subsequently be converted to a diffusion coefficient (*D*).^11^, ^12^ We have previously characterized the diffusional characteristics of agonist and antagonist‐occupied A_3_AR receptor in FCS studies using fluorescent agonists and antagonists and shown that it can be used to distinguish between different receptor affinity states.^13^, ^14^

According to the ternary complex model of GPCR activation proposed in 1980, GPCRs can exist in resting (R) or activated (R*) conformations, the latter of which may also be coupled to a G protein (R*G) with agonists binding preferentially, and with high affinity, to the active conformations.^15^ In 1993, it was found that replacement of the third intracellular loop of the β_2_ adrenergic receptor with the equivalent sequence of the α_1B_ adrenergic receptor resulted in a receptor that signaled in the absence of ligand. To account for this the authors proposed the extended ternary complex model to take into account receptor that was active in the absence of ligand.^16^ A number of GPCRs have been found to naturally exist in a constitutively active form that spontaneously couples to G proteins and activates cell‐signaling pathways in the absence of agonists^17^ and as mentioned above, biophysical data has highlighted the ability of GPCRs to exist in a number of active and inactive states.^5^, ^6^ To increase the levels of constitutive activity, mutations can be introduced that often disrupt the intracellular network of salt bridges stabilized by the conserved DRY (aspartic acid, arginine, and tyrosine) motif found at the bottom of transmembrane domain three.^18^ The A_3_AR has been shown to exhibit low levels of basal constitutive activity and mutation of the highly conserved arginine residue within the DRY motif to alanine (R108A; R3.50A according to the numbering of Ballesteros and Weinstein^19^) has been reported to result in a receptor that displays higher affinity for an agonist radioligand than the wild‐type receptor. In addition, A_3_ARs with this mutation have been shown to have high basal activity in the phospholipase C pathway and reduced basal levels of cAMP, consistent with increased G_i_ activity.^20^ The A_3_AR is a predominately G_i_‐coupled receptor, which leads to inhibition of the cAMP pathway via the G_αi_ subunit and activation of PLC via the βγ subunits.^2^

To further examine the interplay between active and inactive forms of a GPCR, the aim of this study was to introduce the R108A mutation into the A_3_AR and to fully characterize its pharmacology. Using a combination of second messenger assays and FCS, we have studied the signaling, diffusion, and organization of this mutant receptor at the cell surface under a variety of different conditions, including its agonist and β‐arrestin bound states.

## 2 MATERIAL AND METHODS

### 2.1 Generation of constructs used

Previously described A_3_AR fused in‐frame with YFP (A_3_‐YFP), the C‐terminal portion of YFP (A_3_‐vYc)^21^ or GFP (A_3_‐GFP)^14^ on the A_3_AR C‐terminus were used as the template to generate A_3_ R108A‐YFP, A_3_ R108A‐vYc, and A_3_ R108A‐GFP using the QuikChange site‐directed mutagenesis kit (Agilent Technologies, Cheshire, UK). Introduction of the mutation was confirmed by DNA sequencing following by excision of the full length A_3_ R108A‐YFP, A_3_ R108A‐vYc, or A_3_ R108A‐GFP, which was then subcloned into a native pcDNA3.1 vector.

### 2.2 Cell culture and generation of stable cell lines

CHO‐K1 cells stably expressing a cAMP response element‐secreted placental alkaline phosphatase (CRE‐SPAP) reporter gene (CHO CRE‐SPAP) under hygromycin selectivity, CHO‐K1 cells stably expressing β‐arrestin2‐vYnL (CHO β‐arrestin2‐vYnL, a gift from Dr N. Holliday, University of Nottingham, Nottingham, UK; β‐arrestin2 is also known as arrestin3), and CHO‐K1 cells were maintained in DMEM/F12 medium containing 10% of FCS and 2 mM of L‐glutamine at 37°C in a humidified atmosphere of air/CO_2_ (19:1). A_3_‐YFP CRE‐SPAP, CHO β‐arrestin2‐vYnL co‐expressing A_3_‐vYc, and CHO A_3_‐GFP were as previously described.^14^, ^21^ A_3_ R108A‐YFP CRE‐SPAP, CHO β‐arrestin‐2‐vYnL/A_3_ R108A‐vYc, and CHO A_3_ R108A‐GFP cell lines were generated and dilution cloned as described previously.^21^

### 2.3 cAMP accumulation assay

A_3_‐YFP or A_3_ R108A‐YFP CRE‐SPAP cells were grown to confluency in 24‐well plates. On the day of analysis, cells were labeled with [^3^H]‐adenine (2 µCi/mL) in a total volume of 600 µL normal growth medium per well for 2 hours at 37°C/5% CO_2_. After 2 hours, the medium was removed; cells were washed once in serum‐free medium; and fresh serum‐free medium, containing 10 µM rolipram, was added. Increasing concentrations of NECA were added to the required wells and cells incubated for 10 minutes at 37°C. After 10 minutes, 10 µM forskolin (FSK) was added to all wells, apart from basal wells, and cells incubated for a further 1 hour at 37°C. The assay was terminated by the addition of 50 µL concentrated HCl per well. The levels of [^3^H]‐cAMP were measured by sequential Dowex and alumina chromatography and the efficiency of each column determined by the recovery of [^14^C]‐cAMP as described by previously.^22^

### 2.4 CRE‐SPAP gene transcription assay

A_3_‐YFP or A_3_ R108A‐YFP CRE‐SPAP cells were grown to confluency in 96‐well plates. One day before assay, normal growth medium was removed from the cells and replaced by serum‐free medium. Where applicable, pertussis toxin (PTx; 100 ng/mL) was added at this stage. On the day of analysis, the medium was removed and replaced with fresh serum‐free medium containing the required concentration of agonist or antagonist and cells incubated for 1 hour at 37°C/5% CO_2_. After 30 minutes, the required concentration of FSK was added, and cells were incubated for a further 5 hours at 37°C/5% CO_2_. Following this, all medium was removed, 40 µL of fresh serum‐free medium was added to each well and cells incubated for a further 1 hour at 37°C/5% CO_2_. The plates were then incubated at 65°C for 30 minutes to destroy any endogenous alkaline phosphatases. Plates were cooled to room temperature and 100 µL of 5 mM 4‐nitrophenyl phosphate in diethanolamine‐containing buffer [10% (v/v) diethanolamine, 280 mM NaCl, 500 µM MgCl_2_, pH 9.85] was added to each well. The plates were then incubated at 37°C for 25 minutes and the absorbance at 405 nm was measured using a Dynex MRX plate reader (Chelmsford, MA, USA).

### 2.5 ERK1/2 phosphorylation assay

A_3_‐YFP or A_3_ R108A‐YFP CRE‐SPAP cells were grown to confluence in clear 96‐well plates. Where required, cells were treated with 100 ng/mL PTx for 16 hours in normal growth medium. Normal growth medium was replaced with serum‐free medium (DMEM/F12 containing 2 mM L‐glutamine) for a least 2 hours prior to agonist stimulation. Levels of ERK1/2 phosphorylation were measured using the AlphaScreen or AlphaLISA SureFire p‐ERK assay kit (PerkinElmer). For the AlphaScreen assay, cells were stimulated with 10 µM NECA for between 5 and 60 minutes in fresh serum‐free medium or with increasing concentration of NECA for 5 minutes. Medium was removed from each well and replaced with 40 µL SureFire lysis buffer. After shaking for 5 minutes, a 1:80:20:180 v/v dilution of AlphaScreen beads: lysate: activation buffer: reaction buffer in a 5.5 µL total volume was transferred to a white opaque 384‐well proxiPlate in low‐light conditions. After 2 hours of incubation in the dark at room temperature, the fluorescence signal was measured with an EnVision plate reader (PerkinElmer) using standard AlphaScreen settings. For the AlphaLISA assay, cells were stimulated with increasing concentrations of NECA for 5 minutes. Medium was removed and replaced with 50 μL AlphaLISA lysis buffer. After shaking for 10 minutes, 4 μL of lysate was transferred to a white opaque 384‐well proxiPlate, and then, 2 μL of a 1:2:23.5:23.5 v/v dilution of AlphaScreen acceptor beads: activation buffer:reaction buffer1:reaction buffer2 was added to each well and incubated in the dark for 1 hour at room temperature. After 1 hour, 2 μL of 1:49 v/v dilution of Alphascreen donor beads:dilution buffer was added to each well and incubated in the dark for a further 1 hour at room temperature. The fluorescence signal was then measured on a PHERAstar plate reader (BMG Labtech) using standard AlphaScreen settings.

### 2.6 Confocal imaging

Live‐cell imaging was performed on cells grown in Nunc LabTek 8‐well plates and images obtained using a Zeiss LSM710 confocal microscope (Carl Zeiss GmbH, Jena, Germany) fitted with a 63x plan‐Apochromat NA1.4 DIC oil‐immersion objective. A 488 nm argon laser was used to excite both YFP and complemented vYFP (from vYc and vYnL^23^; and emission was detected using a BP505‐530 filter. Normal growth medium was replaced with HEPES‐buffered saline solution (HBSS; 10 mM HEPES, 10 mM D‐glucose, 145 mM NaCl, 5 mM KCl, 1 mM MgSO_4_, 2 mM sodium pyruvate, 1.3 mM CaCl_2_, and 1.5 mM NaHCO_3_) containing 10 µM of NECA where required and cells incubated at 37°C for 60 minutes. A pinhole of 1 Airy Unit was used in all experiments and laser power, gain, and offset were optimized on a per experiment basis and kept consistent between the wild‐type and mutant constructs to allow comparison of fluorescence intensity.

### 2.7 Automated imaging of receptor internalization

CHO β‐arrestin2‐vYnL cells expressing either A_3_‐vYc or A_3_ R108A‐vYc were seeded into the central 60 wells of a clear‐bottomed, black‐walled 96‐well plates (µclear base, Greiner Bio One, Stonehouse, UK) and grown to confluency. Immediately before experimentation, normal growth medium was replaced with serum‐free medium and increasing concentrations of NECA, and cells were incubated for 60 minutes at 37°C/5% CO_2_/95% air. After 60 minutes, all medium was removed and cells were washed once in phosphate‐buffered saline (PBS). Cells were then fixed by the addition of 3% paraformaldehyde solution in PBS for 20 minutes at room temperature. After fixation, cells were washed twice in PBS, before staining of the cell nuclei with the cell permeable dye H33342 (2 µg/mL in PBS) for 20 minutes at room temperature, followed by two additional washes with PBS. Images were obtained using an ImageXpress Ultra confocal plate reader (Molecular Devices, Sunnyvale, CA, USA). Four central images were obtained per well using a Plan Fluor 40x NA0.6 extra‐long working distance objective. vYFP images were obtained by excitation with a 488 nm laser line with emission collected through a 525‐550 nm band‐pass filter and H33342 images obtained by excitation with a 405 nm laser line and emission collected through a 447‐460 nm band‐pass filter. Granularity analysis was performed on the resulting images using a granularity algorithm within MetaXpress software (Molecular Devices) and intensity above background was set for each individual experiment.^23^ Areas of internalized receptors were defined as having a diameter of between 7 and 15 µm and nuclei as having a diameter of between 6 and 9 µm, resulting in a measurement of granule count per cell for each image.

### 2.8 Fluorescence correlation spectroscopy

CHO cells stably expressing A_3_‐GFP or A_3_ R108A‐GFP or CHO β‐arrestin2‐vYnL cells stably co‐expressing either A_3_‐vYc or A_3_ R108A‐vYc were seeded into Nunc LabTek 8‐well plates. On the day of experimentation, cells were washed twice with HBSS prior to ligand stimulation.

FCS measurements were performed using a Confocor2 fluorescence correlation spectrometer (Zeiss) fitted with a c‐Apochromat x40, 1.2 NA water immersion objective. A 488 nm argon laser was used to excite GFP or vYFP (for GFP or BiFC‐tagged cells) and a 633 nm HeNe laser was used to excite the BODIPY 630/650 labeled ABEA‐X‐BY630.^24^ Emission was collected through a 505/550nm bandpass filter and a 650nm longpass filter respectively. For each FCS measurement, fixed camera exposure times were used across all cell lines to ensure cells of comparable fluorescence intensities were selected.

The detection volume was positioned in the x‐y plane above the cell nucleus and subsequently in the z plane using an intensity scan to identify the upper membrane of the cell. For FCS measurements using fluorescent ABEA‐X‐BY630, fluorescence fluctuations were recorded following a 10 seconds prebleaching step at a laser power of 0.2 kW/cm^2^ with two 30 seconds reads recorded using a laser power of 0.3 kW/cm^2^. For measurements using GFP‐tagged receptors, fluorescence fluctuations were recorded following a 10 seconds pre‐bleaching step at a laser power of 0.05 kW/cm^2^ with two 30 seconds reads recorded using a laser power of 0.15 kW/cm^2^.

Fluorescence fluctuations were analyzed using standard autocorrelation analysis within the Zeiss AIM 4.2 software as described in Corriden et al.^14^

For all experiments using GFP‐tagged receptors in conjunction with the fluorescent agonist ABEA‐X‐BY630, cells were washed twice with HBSS. Cells were then treated with 1, 2.5, or 5 nM ABEA‐X‐BY630 (in HBSS) for 10 minutes at 22°C, prior to FCS measurements being recorded from individual cells (22°C). To ascertain the effect of NECA pretreatment on ABEA‐X‐BY630 binding, cells were preincubated with 10 nM NECA (in HBSS) for 10 minutes at 22°C, followed by addition of 2.5 nM ABEA‐X‐BY630 for a further 10 minutes at 22°C. FCS recording were then acquired from individual cells (at 22°C). For all measurements acquired in the presence of ABEA‐X‐BY630, autocorrelation curves were fitted using a model containing one 3D component (*τ_D_*_1_, representing freely diffusing fluorescent ligand) and two 2D diffusion components (*τ_D_*_2_ and *τ_D_*_3_, representing receptor bound ABEA‐X‐BY630) in addition to a pre‐exponential term accounting for the fluorophore triplet state as previously described.^14^ ABEA‐X‐BY630 binding was determined using the value of *N* obtained from the fitted autocorrelation curve and contribution of *τ_D_*_2_ and *τ_D_*_3_ components. The value for *τ_D_*_1_ was fixed during fitting to that determined for free ligand in HBSS. ABEA‐X‐BY630 binding was represented by the *τ_D_*_3_ component alone.^14^

For experiments using cells expressing GFP‐tagged receptors in the absence of ABEA‐X‐BY630, cells were washed twice with HBSS. Cells were then treated with vehicle of NECA (10 μM; 30 minutes at 37°C). Assay plates were placed on the microscope stage and allowed to equilibrate for 5 minutes. FCS measurements were then acquired from individual cells at 22°C. Autocorrelation curves were fitted to a model containing two 2D diffusion components and a pre‐exponential term accounting for GFP photophysical effects. These experimental conditions and autocorrelation model were also used for cells expressing A_3_‐vYc/βarrestin2vYnL or A_3_ R108A‐vYc/βarrestin2vYnL BiFC constructs.

Prior to all experiments, the system was calibrated by calculating the mean dwell time of aqueous solutions of Rhodamine 6G (for the 488 nm laser line; Invitrogen, *D* 2.8 × 10^–6^ cm^2^ s^–1^) or Cy5 NHS ester (for the 633 nm laser; Sigma Aldrich, *D* 3.16 × 10^–6^ cm^2^ s^–1^) fitted using a model containing a single 3D diffusing component with a pre‐exponential triple state component. This allowed the radius of the confocal volume at the beam waist to be determined (ω_1_ = (4⋅*τ_D_* ⋅*D*)^1/2^) for each individual experiment. Average ω_1_ values were subsequently used to calculate beam area at the waist (*A* = *π* · *ω*_1_^2^) and the particle densities (*N*/μm^2^) of fluorescent components present. Average dwell times (*τ_D_*) were converted to diffusion coefficients (*D*) using the equation *D* = *ω*_0_^2^/4.*τ_D_*. Particle number (N) was determined as the fractional contribution of the *τ_D_*_2_ (GFP or BiFC experiments) or *τ_D_*_3_ (ABEA‐X‐BY630) diffusing component multiplied by the total particle number (*N*), determined from the autocorrelation curve fit. Particle number was subsequently expressed as particles per μm^2^ (N/μm^2^).

Molecular brightness (*ε*) values were determined from photon counting histogram (PCH) analysis of the fluctuation data obtained in FCS experiments. For each individual experiment, first‐order correction values were obtained from Rhodamine 6G calibration data (20 nM) fitted to a one component fit using a bin time of 20 μs. This first‐order correction value was used for all subsequent data fitting. For GFP‐tagged receptors, PCH data were fitted using a bin time of 100 μs with all traces preferentially fitting to a one component fit. For BiFC receptor/βarrestin‐2 complexes all traces preferentially fit to a PCH model containing two components using a bin time of 100 μs (brightness 1 and brightness 2).

### 2.9 Data analysis

For [^3^H]‐cAMP experiments, data were normalized to basal (in the absence of agonist) and 10 µM FSK response. For ERK1/2 phosphorylation and internalization assays, data were normalized to basal and the maximal 10 µM NECA response in wild‐type cells, which was included in each separate experiment.

All data were fitted using nonlinear regression in Prism 7 (GraphPad Software, San Diego, CA, USA). Concentration response curves were fitted to the following equation:

$$\text{Response}=\frac{E_{\max}\times \left[A\right]}{\left[A\right]+{\text{EC}}_{50}}$$

where *E*_max_ is the maximal response and the EC_50_ is the molar concentration of agonist required to generate 50% of the *E*_max_.

For FCS experiments, data from curve fitting were analyzed in Microsoft Excel, and then, representation and statistical analysis were performed using GraphPad Prism 8.

Data are presented as mean ± SEM with the number of individual cells and/or independent experiments stated. Statistical significance was determined using unpaired Student's t test or one‐way analysis of variance (ANOVA) with post hoc Tukey's multiple comparisons analysis as stated.

## 3 RESULTS

### 3.1 A_3_ R108A binds agonists with high affinity

Previous studies have found that substitution of arginine for alanine at residue 108 in the A_3_AR resulted in a receptor that was constitutively active.^20^ In the present study, we have used a combination of microscopy and second messenger assays to evaluate the impact of this mutation on agonist binding, signaling, internalization, and diffusion characteristics.

**FIGURE 1:**
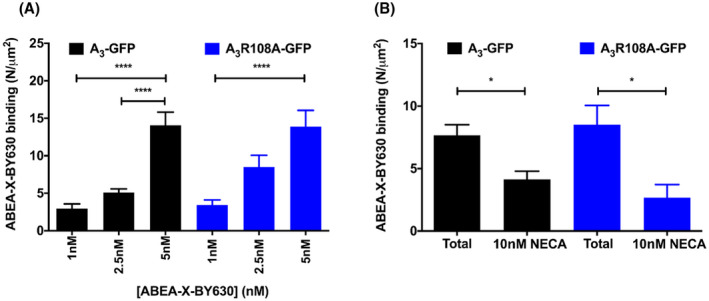
High‐affinity agonist binding measured by FCS. CHO cells stably expressing A_3_‐GFP (black bars) or A_3_ R108A‐GFP (blue bars) were stimulated with 1, 2.5, or 5 nM fluorescent adenosine agonist ABEA‐X‐BY630 (22°C; 10 minutes; A). Total ABEA‐X‐BY630 binding at each concentration was measured using fluorescence correlation spectroscopy (FCS). Data were collected from four independent experiments (n = 32‐34 cells) and expressed as mean ± SEM. Statistical significance was determined using a one‐way ANOVA with Tukey's multiple comparisons test (****P < .0001). B, To confirm ABEA‐X‐BY630 binding at the A_3_ or A_3_ R108A receptor, cells were incubated with 10 nM NECA (22°C; 10 minutes) followed by 2.5 nM ABEA‐X‐BY630 (22°C; 10 minutes). Data were acquired from three independent experiments (n = 10‐14 cells) and are expressed as mean ± SEM Statistical significance of ABEA‐X‐BY630 displacement at each receptor was determined using unpaired Student's t tests (*P < .05)

To investigate if A_3_AR R108A can still bind agonists with high affinity and express at the cell surface, FCS was used to monitor the binding of a fluorescent agonist, ABEA‐X‐BY630. We have previously shown that the use of very low concentrations of fluorescent agonists can facilitate selective monitoring of the high‐affinity active form (R*) of the receptor.^13^ First, the R108A mutation was introduced into an A_3_‐GFP fusion protein and a stable CHO cell line was generated. Using CHO cells stably expressing A_3_‐GFP^14^ or A_3_ R108A‐GFP and low concentrations of ABEA‐X‐BY630 (1, 2.5, and 5 nM, 10 minutes, 22°C), FCS measurements of the fluctuations resulting from the fluorescent ligand were measured at the upper plasma membrane. For both A_3_‐GFP and A_3_ R108A‐GFP, the number of bound ABEA‐X‐BY630 molecules (N/μm^2^) increased with increasing concentrations of ligand (Figure 1A). The diffusion coefficient (*τ_D_*_3_) of bound ABEA‐X‐BY630 (2.5 nM) was similar in both cell lines (A_3_‐GFP = 0.15 ± 0.02 μm^2^/s; A_3_ R108A‐GFP = 0.16 ± 0.02 μm^2^/s). To further investigate this high‐affinity binding, A_3_‐GFP and A_3_ R108A‐GFP cells were preincubated with a low concentration of the nonselective adenosine receptor agonist NECA (10 nM, 10 minutes, 22°C) prior to the addition of 2.5 nM ABEA‐X‐BY630. A significant decrease in ABEA‐X‐BY630 binding was observed for both A_3_‐GFP and A_3_ R108A‐GFP (Figure 1B *P* < .05; unpaired Student's t tests). As the concentration of NECA added here is very low, this indicates that both receptors can bind agonist with high affinity at the cell surface.

### 3.2 Influence of the R108A mutation in A_3_AR on downstream functional responses

**FIGURE 2:**
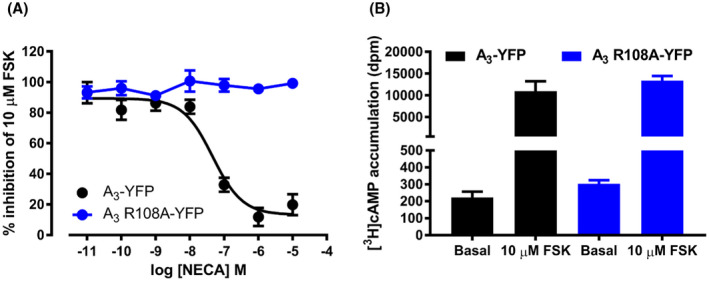
Agonist‐mediated inhibition of FSK‐stimulated cAMP levels by the A_3_‐YFP and A_3_R108A‐YFP receptors. A, A_3_‐YFP (black circles) and A_3_ R108A‐YFP‐expressing (blue circles) cells were loaded with [^3^H]‐adenine, and then, exposed to increasing concentrations of NECA for 10 minutes, followed by the addition of 10 μM FSK for 1 hour. Levels of [^3^H]‐cAMP were estimated by scintillation counting after separation by sequential Dowex and alumina chromatography. Data were normalized to basal (in the absence of FSK) and 10 µM FSK [^3^H]‐cAMP accumulation for each cell line. Data shown represent the mean ± SEM of three experiments performed in triplicate. B, Bar graph of basal and 10 µM FSK [^3^H]cAMP levels in dpm in A_3_‐YFP (black bars) and A_3_ R108A‐YFP (blue bars) expressing cells. Data shown in (A) represent the mean ± SEM of three experiments performed in triplicate and in (B) one representative example of three separate experiments performed in triplicate and data represent the mean ± SEM

To investigate the impact of the R108A mutation on constitutive and agonist‐mediated signaling, the R108A mutation was introduced into an A_3_‐YFP construct and a stable CHO cell line containing a cAMP reporter gene (cAMP response element linked secreted placental alkaline phosphatase; CRE‐SPAP) was generated. The effect of this mutation on cAMP production was investigated by two independent methods: first, by direct measurement of [^3^H]‐cAMP levels and second, by cAMP‐mediated activation of the CRE‐SPAP reporter gene and compared to those obtained in a CHO CRE‐SPAP cell line expressing wild‐type A_3_‐YFP. As the A_3_AR predominantly couples to the G_i_ family of G proteins, activation of the receptor results in an inhibition of forskolin (FSK)‐stimulated cAMP production. As expected, in A_3_‐YFP cells, stimulation with the known A_3_AR agonist NECA for 1 hour caused a substantial concentration‐dependent inhibition of FSK‐stimulated [^3^H]‐cAMP accumulation (Figure 2A, Table 1). In A_3_ R108A‐YFP cells, NECA did not induce any measurable inhibition of cAMP production (Figure 2A). In addition, there was no difference in the basal (*P* = .26, unpaired t test) or FSK‐stimulated (*P* = .84, unpaired t test) levels of cAMP produced in A_3_ R108A‐YFP‐expressing cells compared to cells expressing A_3_‐YFP, suggesting that A_3_ R108A‐YFP does not constitutively couple to G_i_ (Figure 2B).

**TABLE 1:** Summary of pEC_50_ values and relative efficacy of NECA at A_3_‐YFP and A_3_ R108A‐YFP in second messenger assays

	cAMP accumulation assay			CRE‐SPAP assay			pERK1/2 assay		
	pEC_50_	Maximum response (% inhibition of 10 µM FSK)	n	pEC_50_	Maximum response (% inhibition of 3 µM FSK)	n	pEC_50_	Maximum response (% 10 µM NECA)	n
A_3_‐YFP	7.34 ± 0.04	80.2 ± 6.8	3	7.41 ± 0.09	68.3 ± 9.1	6	7.93 ± 0.13	100	12
A_3_ R108A‐YFP	NR	No inhibition	3	NR	No inhibition	6	ND	4.9 ± 2.0	15

Note

Values are mean ± SEM from n separate experiments. NR = no response. ND = not determined due to inconsistent and small size of the response. Maximum response in pERK1/2 assay is the response in each cell line relative to that of 10 µM NECA in A_3_‐YFP‐expressing cells. In CRE‐SPAP and ^3^H‐cAMP assays, maximal response is the maximal inhibition of FSK‐stimulated SPAP production or ^3^H‐cAMP accumulation achieved with each agonist in each cell line. Values are mean ± SEM from n separate experiments

**FIGURE 3:**
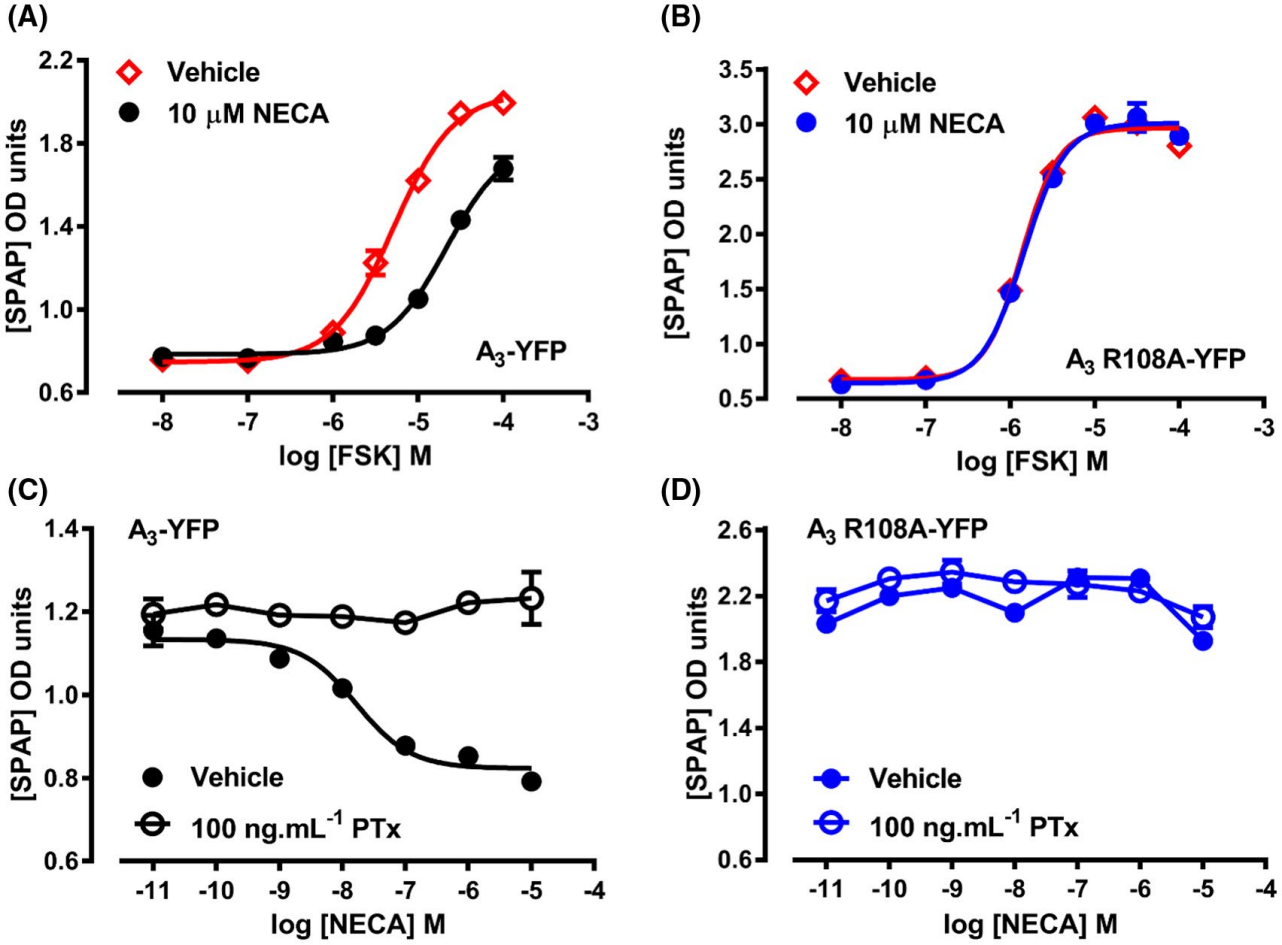
Agonist and PTx effects on FSK‐stimulated CRE‐mediated SPAP production by the A_3_‐YFP and A_3_ R108A‐YFP receptors. A_3_‐YFP (A) and A_3_ R108A‐YFP‐expressing (B) cells were pretreated with 10 µM NECA (30 minutes, circles) prior to the addition of increasing concentrations of FSK (no pretreatment, open diamonds) and the levels of SPAP produced monitored. A_3_‐YFP (C) and A_3_ R108A‐YFP‐(D) expressing cells were treated overnight with normal medium (closed circles) or medium containing 100 ng/mL PTx (open circles) prior to treatment with increasing concentrations of NECA and levels of SPAP produced monitored. Data shown represent mean ± SEM of one experiment performed in triplicate and are representative of four performed

To further investigate the ability of A_3_ R108A to signal through the cAMP pathway, the ability of the receptor to activate CRE‐mediated gene transcription was investigated. Initially, the effect of a fixed concentration of the agonist NECA (10 µM, 30 minutes) on the potency of FSK was tested. In A_3_‐YFP‐expressing cells, there was a rightward shift in the FSK concentration response curve due to the inhibition of FSK‐stimulated CRE‐mediated SPAP production (Figure 3A, pEC_50,_ FSK = 5.27 ± 0.13; FSK + 10 μM NECA = <4; n = 5). In contrast, in A_3_ R108A‐YFP, there was no change in the pEC_50_ of FSK in the presence of NECA (Figure 3B, pEC_50,_ FSK = 5.54 ± 0.11; FSK + 10 μM NECA = 5.50 ± 0.10; n = 5). The potency of NECA to inhibit FSK‐stimulated CRE‐mediated SPAP production was then determined (Figure 3C,D). As seen in the [^3^H]‐cAMP assay, in the CRE‐SPAP assay NECA was unable to stimulate any reduction in FSK‐stimulated SPAP production in A_3_ R108A‐YFP‐expressing cells. Both cell lines were then treated with Pertussis toxin (PTx) to block any agonist mediated and constitutive signaling to G_i/o_ G proteins. As expected with a predominantly G_i/o_‐coupled receptor, PTx treatment abolished the ability of NECA to inhibit FSK‐stimulated SPAP production in A_3_‐YFP cells. In A_3_ R108A‐YFP‐expressing cells there was no effect on SPAP production upon PTx treatment, supporting the evidence that A_3_ R108A does not show constitutive activity through the cAMP pathway and no coupling to G_s_ (Figure 3D).

**FIGURE 4:**
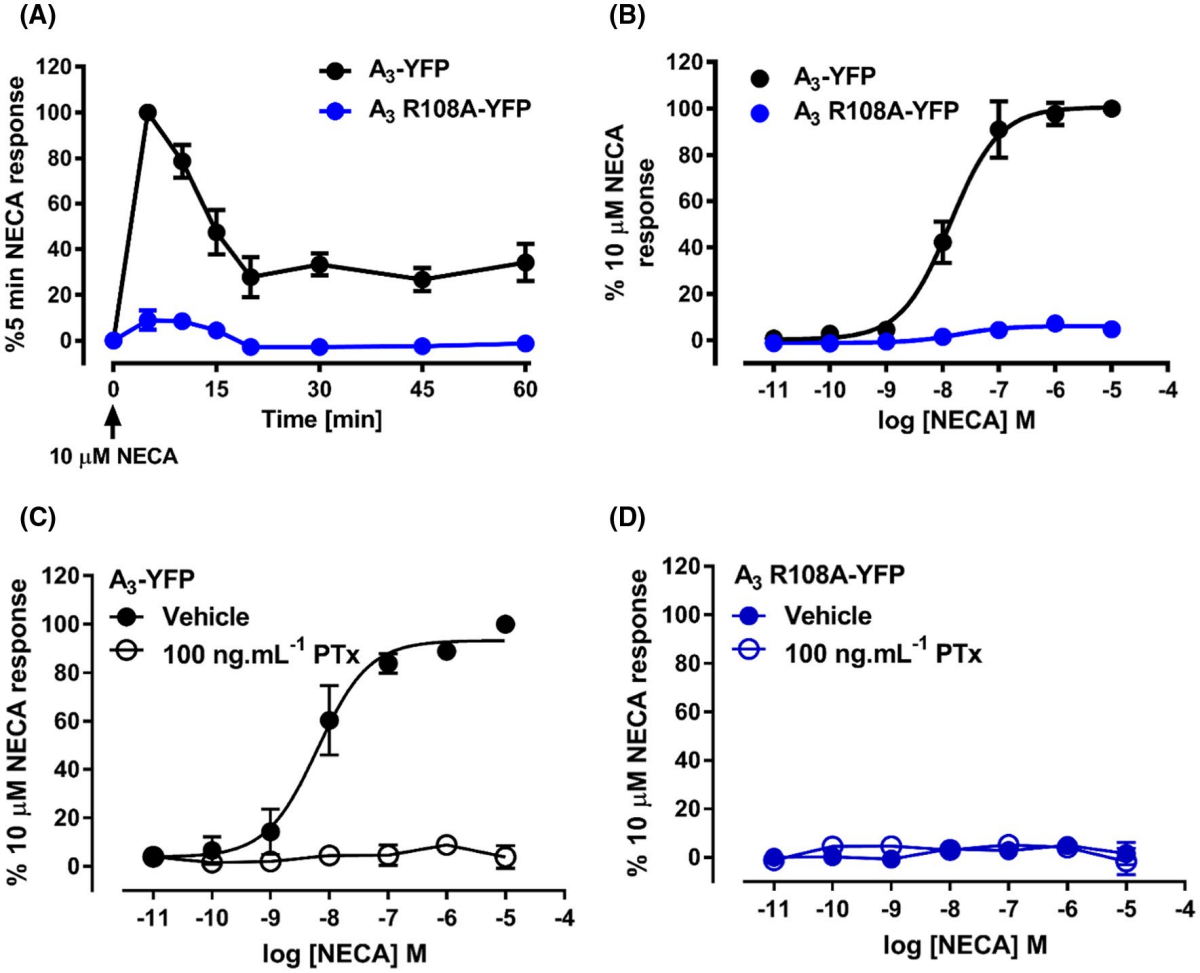
Agonist‐mediated phosphorylation of ERK1/2 by the A_3_‐YFP and A_3_ R108A‐YFP receptors. A_3_‐YFP (black circles) and A_3_ R108A‐YFP (blue circles) expressing cells were exposed to 10 µM NECA for increasing amount of time (A) or to increasing concentrations of NECA for 10 minutes (B). Levels of phosphorylated ERK1/2 were quantified using the SureFire AlphaScreen kit. Data were normalized to the 10 µM NECA response in A_3_‐YFP‐expressing cells. The data shown represent the mean ± SEM of nine (B, A_3_ R108A‐YFP), six (B, A_3_‐YFP), or four (A) experiments performed in triplicate. A_3_‐YFP (C) and A_3_ R108A‐YFP (D) expressing cells were treated overnight with normal medium (closed circles) or medium containing 10 ng/mL PTx (open circles) prior to treatment with increasing concentration of NECA for 5 minutes. Levels of phosphorylated ERK1/2 were quantified using the SureFire AlphaLISA kit. Data were normalized to the 10 μM NECA response in A_3_‐YFP‐expressing cells. The data shown represent the mean ± SEM of six (C and D) experiments performed in triplicate

The A_3_AR has been shown to signal through a variety of other pathways, including the ERK1/2 pathway. The effect of the R108A mutation on the time‐dependence of the A_3_AR to stimulate ERK1/2 phosphorylation was determined. Stimulation of A_3_‐YFP with NECA (10 µM) resulted in an increase in the levels of phosphorylated ERK1/2 that was maximal at 5 minutes (Figure 4A). In NECA‐treated A_3_‐YFP cells, the levels of pERK1/2 reduced to a plateau of approximately 40% of the maximal response, which was sustained for the duration of the experiment (60 minutes pERK = 34 ± 8% of maximal, n = 4). Unlike the cAMP assays, in the A_3_ R1018A‐YFP‐expressing cells, NECA appeared to stimulate a small increase in levels of pERK1/2. The peak response at A_3_ R108A‐YFP was after 5 minutes, with 7.3 ± 1.2% of the maximal A_3_‐YFP response. As in these experiments a maximal concentration of agonist was used, the concentration dependence of the response was then measured (Figure 4B–D). Stimulation of A_3_‐YFP cells with increasing concentrations of NECA for 5 minutes resulted in a large increase in pERK with pEC_50_ of 7.74 ± 0.21 (n = 12) (Figure 4B,C). In contrast, there was no consistent concentration dependence of this small response to NECA in cells expressing A_3_ R108A‐YFP (Figure 4B,D). To determine if A_3_‐YFP‐stimulated ERK phosphorylation was mediated by G_i/o_ protein activation, cells were pretreated with PTx (100 ng/mL/16 hours) prior to stimulation with NECA. In A_3_‐YFP cells, PTx abolished the ability of the receptor to stimulate ERK phosphorylation indicating that it is fully dependent on G protein activation (Figure 4C). In A_3_ R108A‐YFP‐expressing cells, there was no change in the pERK response after PTx treatment (Figure 4D).

### 3.3 Membrane distribution and diffusion characteristics of A_3_ and A_3_ R108A in response to agonist treatment

**FIGURE 5:**
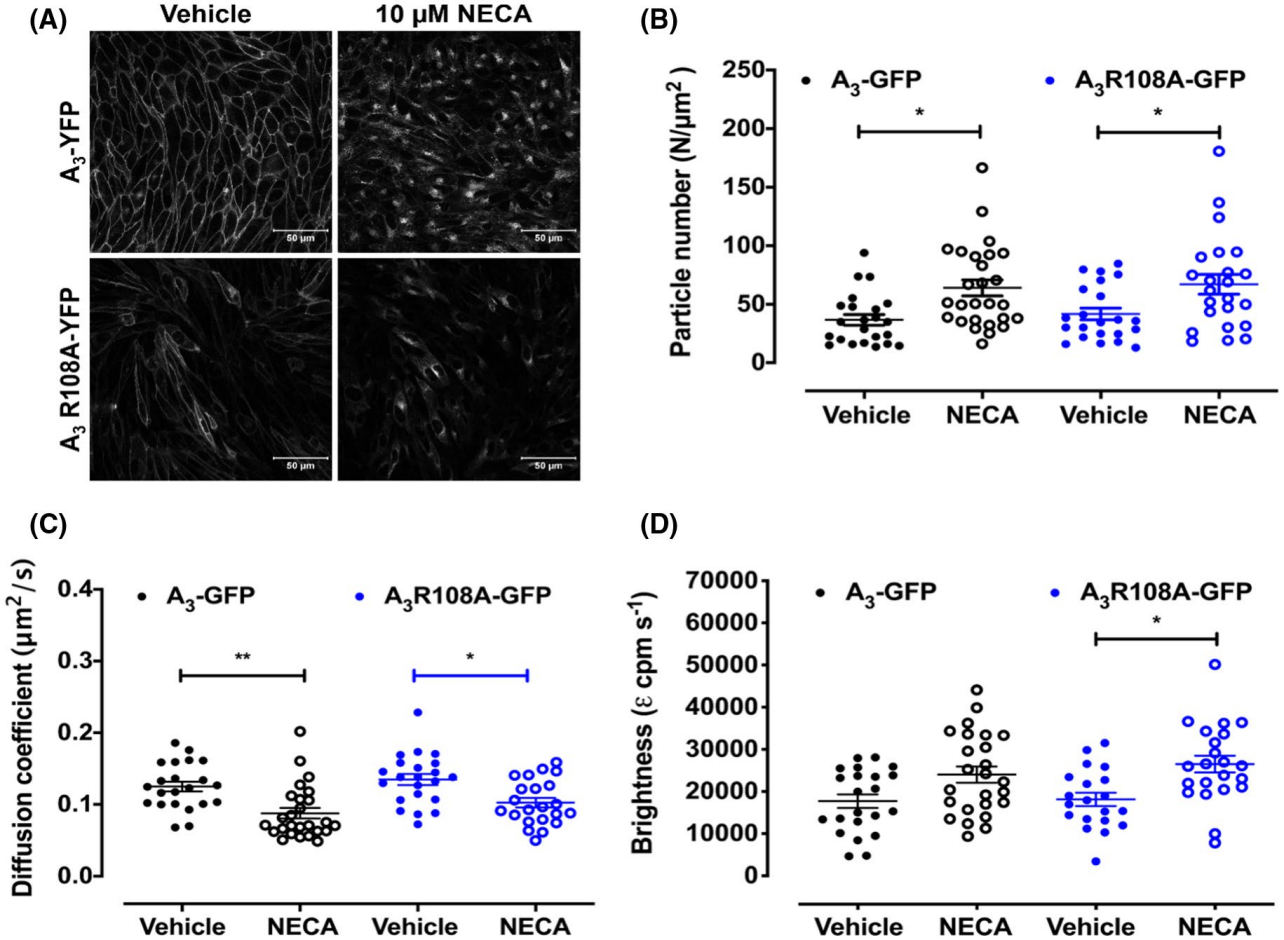
Agonist‐stimulated internalization and reorganization of A_3_‐YFP and A_3_ R108A‐YFP. A, Confocal images of A_3_‐YFP (top panels) and A_3_ R108A‐YFP‐ (bottom panels) expressing cells were obtained in the absence of agonist (left‐hand panels) and after treatment with 10 µM NECA for 60 minutes at 37°C (right‐hand panels). Images are representative of those obtained in three separate experiments. For fluorescence correlation spectroscopy (FCS) experiments, CHO cells stably expressing A_3_‐GFP or A_3_ R108A‐GFP were stimulated with vehicle or NECA (10 μM; 37°C for 30 minutes). Particle numbers (B) and diffusion coefficients (C) of A_3_‐GFP (black symbols) or A_3_ R108A‐GFP (blue symbols) were determined from data acquired from three independent experiments (n = 21‐26 cells) and are expressed as mean ± SEM. Molecular brightness (D) values were determined from FCS traces using photon counting histogram analysis (PCH) with all traces fitting to a one component model. Statistical significance was determined using one‐way ANOVA with Tukey's multiple comparisons test (*P < .05; **P < .01)

We next investigated the cellular distribution of the mutant receptor and determined whether this changed in response to agonist treatment. Initial confocal imaging confirmed the ABEA‐X‐BY630‐binding data showing that A_3_ R108A‐YFP was predominately expressed at the cell surface. After treatment of A_3_ R108A‐YFP with a saturating concentration of NECA, there was a distinct change in the distribution of the receptor from the cell surface to intracellular granules (Figure 5A). This is similar to the pattern observed with A_3_‐YFP, although the intracellular granules in A_3_‐YFP appeared more defined. The less well‐defined granules of internalized receptor in agonist‐treated A_3_ R108A‐YFP‐expressing cells made it difficult to quantify the numbers of granules using automatic image analysis as we have previously performed for A_3_‐YFP.^21^

To further investigate the redistribution of wild‐type and R108A versions of the A_3_AR, FCS studies were performed on A_3_‐GFP and A_3_ R108A‐GFP cells treated with NECA. In contrast to our initial FCS experiments, which specifically monitored agonist‐receptor complexes, in this set of experiments we monitored the fluctuations of the receptor‐GFP itself. Here, we were therefore quantifying the diffusion coefficient (μm/s^2^), receptor density (particle number, N/μm^2^), and clustering (molecular brightness, *ε*), to gain insights into the diffusion, density, and clustering of the total membrane receptor population. Under basal conditions, the FCS parameters for A_3_‐GFP and A_3_ R108A‐GFP were very similar (Figure 5, Table 2), demonstrating similar expression levels at the cell surface and basal organization for both receptors. For both A_3_‐GFP and A_3_ R108A‐GFP, there was an increase in receptor density (N/μm^2^), slowing in the diffusion (D, μm^2^/s), and increase in receptor aggregation (molecular brightness, *ε*) at the upper plasma membrane upon NECA treatment (Figure 5, Table 2). Taken together, this indicates that there is significant reorganization of both A_3_‐GFP and A_3_ R108A‐GFP upon agonist treatment.

**TABLE 2:** Summary of particle number, diffusion, and molecular brightness for vehicle and agonist‐stimulated A_3_‐GFP and A_3_ R108A‐GFP

		Particle number (N/μm^2^)	Diffusion coefficient (*τ_D_*_2_, μm^2^/s)	Molecular brightness (*ε*_1_, cpm. s^–1^)	n
A_3_‐GFP	Vehicle	36.7 ± 4.6	0.13 ± 0.007	18 111 ± 1582	23
	NECA	64.1 ± 6.8*	0.09 ± 0.007**	24 027 ± 1933	26
A_3_ R108A‐GFP	Vehicle	43.6 ± 5.1	0.13 ± 0.008	18 152 ± 1584	22
	NECA	67.1 ± 8.5*	0.10 ± 0.007*	26 351 ± 1915*	23

Note

Particle number (N/μm^2^), diffusion coefficient (*τ_D_*_2_, μm^2^/s), and molecular brightness (*ε*_1_, cpm. s^–1^) were determined from FCS traces obtained from A_3_‐GFP and A_3_ R108A‐GFP cells in the absence or presence of NECA (10 µM, 30 minutes). Values are mean ± SEM from n separate experiments. * and ** denote significance vs vehicle in the same cell line determined using one‐way ANOVA with Tukey's multiple comparisons test (*P < .05; **P < .01).

### 3.4 Influence of the R108A mutation on the diffusion characteristics of receptor‐arrestin complexes

**FIGURE 6:**
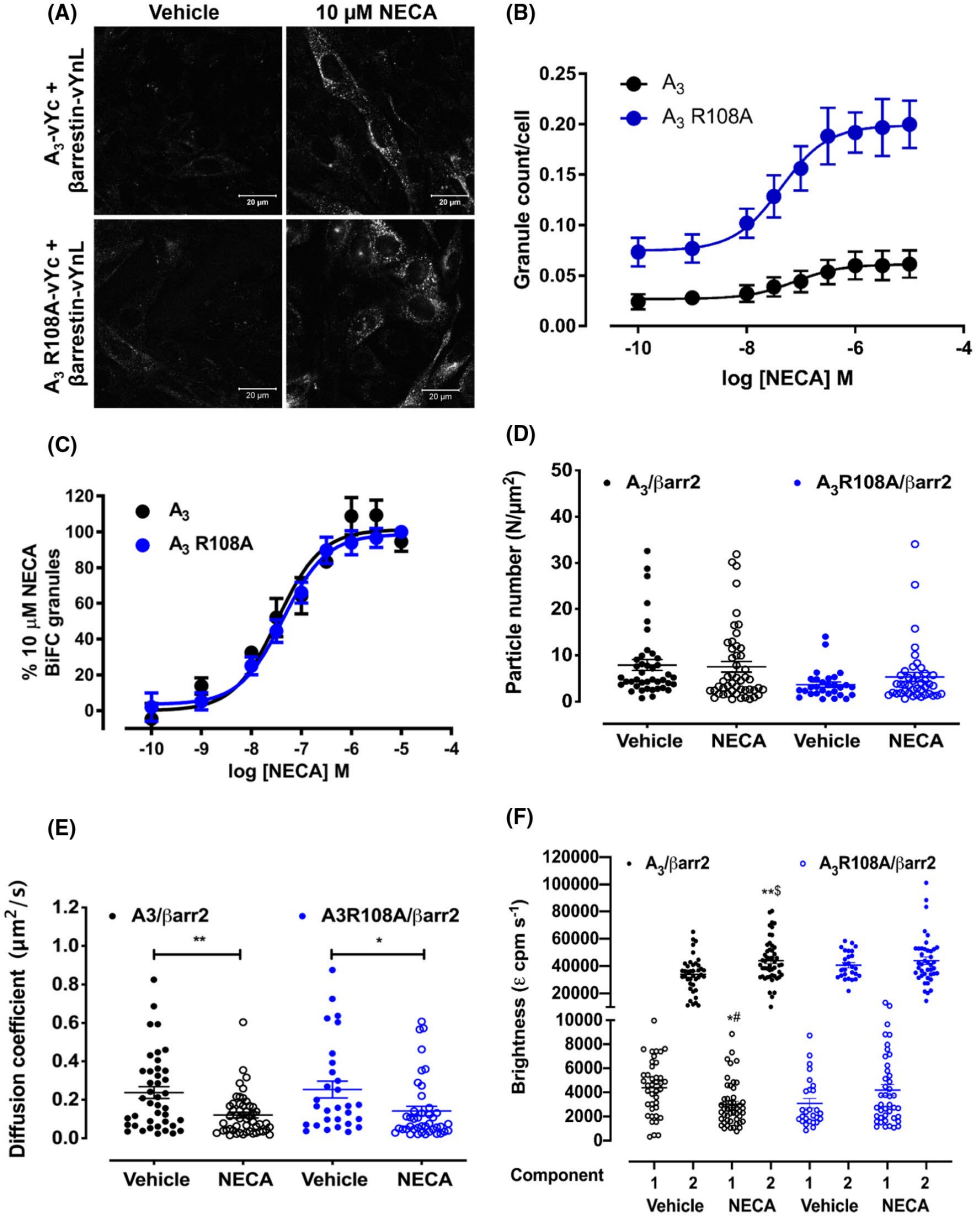
Quantitative analysis of A_3_ and A_3_ R108A β‐arrestin2 BiFC complexes using image analysis and FCS. A, Confocal images of A_3_‐vYc/βarrestin2‐vYnL (top panels) and A_3_ R108A‐vYc/βarrestin2‐vYnL (bottom panels) expressing cells were obtained after treatment with vehicle (left‐hand panels) or 10 µM NECA (right‐hand panels) for 60 minutes at 37°C. Images are representative of those obtained in three separate experiments. B, A_3_‐vYc/βarrestin2‐vYnL (black circles) and A_3_ R108A‐vYc/βarrestin2‐vYnL (blue circles) cells were treated with increasing concentrations of NECA for 60 minutes. Automated confocal images were obtained on the ImageXpress Ultra plate reader and granularity analysis performed to quantify levels of internalized vYFP. The data shown represent (B) granule count per cell and (C) normalized granule count per cell as a percentage of the 10 µM NECA response for each receptor. Each data point represents the mean ± SEM of five experiments performed in triplicate. For fluorescence correlation spectroscopy (FCS) experiments, CHO cells stably expressing A_3‐_vYc/βarrestin2‐YnL (black symbols) or A_3_ R108A‐vYc/βarrestin2‐vYnL (blue symbols) were stimulated with vehicle or NECA (10 μM; 37°C for 30 minutes). Particle numbers (D) and diffusion coefficients (E) were determined from data acquired from three independent experiments (n = 27‐50 cells) and are expressed as mean ± SEM. Molecular brightness (F) values were determined from FCS traces using photon counting histogram analysis (PCH). For each condition, molecular brightness values from the first (open circles) or second (closed circles) component are shown. Traces from all cells fit to a two‐component model of molecular brightness. Statistical significance was determined using one‐way ANOVA with Tukey's multiple comparisons test (*P < .05; **P < .01). *# = comparison of brightness component 1 of vehicle traces with component 1 of NECA traces for A_3_/βarr2vYnL, **$ = comparison of brightness of component 2 of vehicle traces with component 2 of NECA traces for A_3_/βarr2vYnL

In view of the change in the distribution of A_3_ R108A upon agonist treatment, supported by the imaging and FCS data, we next investigated if this mutant receptor could interact with β‐arrestin2 (also known as arrestin3). To do this, we used a bimolecular fluorescence complementation (BiFC) approach to trap receptor‐β‐arrestin complexes through the interaction between two fragments of venus YFP (vYFP) and subsequent visualization of the resulting complemented mature vYFP chromophore.^25^ We have previously^21^ generated a stable CHO cell line expressing β‐arrestin2‐vYnL (residues 1‐173 of vYFP) in combination with A_3_‐vYc fusion protein (residues 155‐238 of vYFP), therefore, we generated an additional CHO cell line co‐expressing β‐arrestin2‐vYnL and A_3_ R108A‐vYnL. Confocal imaging showed low levels of BiFC under control conditions in both A_3_‐vYc/βarrestin2‐vYnL and A_3_ R108A‐vYc/βarrestin2‐vYnL cell lines and as BiFC is essentially irreversible any low levels of receptor‐arrestin interaction would result in trapped BiFC complexes. This suggests that A_3_ R108A shows no increase in basal β‐arrestin2 interaction compared to the wild‐type receptor. Treatment with 10 μM NECA for 60 minutes caused a substantial increase of BiFC fluorescence in both cell lines indicating formation and internalization of receptor‐β‐arrestin complexes (Figure 6A). Quantification of the concentration‐dependent increase in BiFC was carried out using an automated confocal plate reader (MD ImageXpress Ultra) and image analysis (MetaXpress) to detect fluorescent granules of complemented vYFP. A concentration‐dependent increase in BiFC was observed in both cell lines indicating agonist‐dependent recruitment of β‐arrestin2 (Figure 6B,C). When taking into account the granule count per cell, there appears to be a larger number of granules in the A_3_ R108A‐vYc/βarrestin2‐vYnL‐expressing cells compared to A_3_‐vYc/βarrestin2‐vYnL cells. The granularity analysis takes into account the total number of cells in the field of view and as observed in Figure 6A, there is a lower number of A_3_‐vYc/βarrestin2‐vYnL‐expressing cells compared to A_3_ R108A‐vYc/βarrestin2‐vYnL which results in a lower granule count per cell. The potency (pEC_50_) of NECA‐stimulated BiFC in the A_3_ R108A‐vYc/βarrestin2‐vYnL cell line was 7.35 ± 0.21 which was very similar to that in the A_3_‐ vYc/βarrestin2‐vYnL cell line (7.35 ± 0.10; *P* = .98, unpaired t test).

To gain further insights into the effects of the R108A mutant on the ability of the A_3_AR to engage β‐arrestin2, FCS measurements were taken on the upper plasma membrane of cells expressing A_3_‐vYc/βarrestin2‐vYnL or A_3_ R108A‐vYc/βarrestin2‐vYnL. Under basal conditions, FCS measurements were obtained in both cell lines, indicating that both A_3_AR and A_3_AR R108A could recruit and form complexes with β‐arrestin2 in the absence of agonist (Figure 6D, Table 3). In the absence of agonist, there were fewer complexes (lower particle number) in the A_3_AR R108A cells compared to the A_3_AR cells, but no difference in the diffusion coefficient obtained (μm^2^/s) for A_3_‐vYc/βarrestin2‐vYnL complexes compared to A_3_ R108A‐vYc/βarrestin2‐vYnL (Figure 6E, Table 3). Upon agonist stimulation, there was a significant slowing of both A_3_‐vYc/βarrestin2‐vYnL and A_3_ R108A‐vYc/βarrestin2‐vYnL complexes as indicated by the decreased diffusion co‐efficient (Figure 6D) but no significant change in the particle number (Figure 6D). The majority of the photon counting histogram (PCH) traces, which were used to calculate molecular brightness, under both basal and agonist‐stimulated conditions fitted to two components indicating two populations of significantly different brightness (Figure 6F). This contrasted with the A_3_R‐GFP data where only one population was observed (Figure 5D). In A_3_‐vYc/βarrestin2‐vYnL cells, NECA caused a significant increase in the brightness of the second component, whereas in A_3_ R108A‐vYc/βarrestin2‐vYnL cells NECA stimulation did not result in a change in the brightness in either component (Figure 6F, Table 3).

**TABLE 3:** Summary of particle number, diffusion, and molecular brightness for vehicle and agonist‐stimulated A_3_‐vYc/βarrestin2‐vYnL and A_3_ R108A‐vYc/βarrestin2‐vYnL

		Particle number (N/μm^2^)	Diffusion coefficient (*τ_D_*_2_, μm^2^/s)	Molecular brightness (*ε*_1_, cpm s^–1^)		n
				Component 1	Component 2	
A_3_‐vYc/βarrestin2‐vYnL	Vehicle	7.9 ± 1.2	0.24 ± 0.03	4383 ± 359	34 006 ± 2063	40
	NECA	7.5 ± 1.1	0.12 ± 0.02**	2996 ± 255*^#^	44 028 ± 2187**^$^	50
A_3_ R108A‐vYc/βarrestin2‐vYnL	Vehicle	3.7 ± 0.6	0.25 ± 0.04	3087 ± 392	40 618 ± 1892	27
	NECA	5.3 ± 1.0	0.14 ± 0.02*	4185 ± 461	43 968 ± 2712	42

Note

Particle number (N/μm^2^), diffusion coefficient (*τ_D_*_2_, μm^2^/s), and molecular brightness (*ε*_1_, cpm. s^–1^) were determined from FCS traces obtained from A_3_‐vYc/βarrestin2‐vYnL and A_3_ R108A‐vYc/βarrestin2‐vYnL cells in the absence or presence of NECA (10 µM, 30 minutes). Values are mean ± SEM from n separate experiments. * and ** denote significance vs vehicle in the same cell line determined using one‐way ANOVA with Tukey's multiple comparisons test (*P < .05; **P < .01) *^#^ = comparison of brightness component 1 of vehicle traces with component 1 of NECA traces for A_3_‐vYc/βarrestin2‐vYnL, **^$^ = comparison of brightness of component 2 of vehicle traces with component 2 of NECA traces for A_3_‐vYc/βarrestin2v‐YnL.

## 4 DISCUSSION

The organization of GPCRs at the plasma membrane is the focus of much recent attention. Through the use of advanced microscopy techniques, it is clear that some GPCRs are localized to discrete microdomains, and reorganization occurs upon ligand activation.^26^ It has been suggested that differences in membrane organization underpin the difference in signaling outcomes from different ligands.^12^, ^27^ Therefore, understanding the influence of the binding of effector proteins and receptor conformation on its membrane organization is required. To achieve this, we characterized the membrane organization of a mutant (R108A) of the adenosine A_3_ receptor (A_3_AR), which showed impaired G protein coupling but retained the ability to recruit β‐arrestin2.

The residue mutated in this study, R108 or R3.50 according to the numbering of Ballesteros and Weinstein,^19^ is one of the most conserved residues within family A GPCRs, with Arg being present in 95% of receptors.^28^ Many of the conserved residues within GPCR transmembrane domains are involved in the switch between inactive and active receptor conformations. R3.50 is one of three highly conserved residues at the cytoplasmic end of transmembrane helix 3 and, with two adjacent residues, forms the E/DRY motif. In the rhodopsin crystal structure, the DRY motif forms a strong hydrogen bonding network. Furthermore, the interaction between Arg135 and the Glu247 and Thr251 residues in helix 6 has been proposed to form an “ionic lock” holding the receptor in the inactive conformation.^29^ To date, there is no crystal structure of the A_3_AR available, but the closely related adenosine A_2A_ receptor (A_2A_ AR) crystal structure has been solved bound to an engineered G_s_ protein (mini‐G_s_). Within this structure, R3.50 undergoes a rotamer change when compared to the agonist‐bound intermediate structure and also forms van der Waals interactions with the mini‐G_s_.^30^ Therefore, it is no surprise that mutation of this residue in the A_3_AR impairs G protein coupling as we have observed in this study. We examined the ability of A_3_AR R108A to inhibit the cAMP pathway by two separate methods and to stimulate phosphorylation of ERK. In all three assays, no consistent concentration‐dependent responses to NECA were observed. The data obtained within the FCS experiments indicated that A_3_AR R108A still retained the ability to bind agonist with high affinity and, taken together with the functional and structural data from the A_2A_AR, this suggests that there is a disconnect between agonist binding and effective G protein coupling.

Within the current study, we found no evidence for constitutive second messenger signaling or internalization of A_3_AR R108A. This is in contrast to a previous study on the A_3_AR, which found that in COS‐7 (an African green monkey kidney fibroblast like cell line), A_3_AR R108A displayed lower basal cAMP levels, higher basal inositol phosphate formation, and an increase in affinity for radiolabeled agonist.^20^ In the present study, we used CHO (Chinese Hamster Ovary) cells and did not see any decrease in basal cAMP levels in the [^3^H]‐cAMP accumulation assay, therefore, these difference may be due to the different cell backgrounds and endogenously expressed proteins within these cells. In the CRE‐SPAP assay, basal levels of activation are difficult to compare across cell lines. To address this, we determined the potency of FSK, as any constitutive activity would lead to a lower potency (similar to the effect observed in the presence of NECA in wild‐type cells). The potency of FSK in A_3_AR R108A‐expressing cells was similar to that in A_3_AR cells, indicating no constitutive activation through the cAMP pathway. Mutation of the highly conserved DRY residues in Family A GPCRs has a range of effects although in many cases it is mutation of the aspartic acid (D) that results in constitutive activity^31^ and this been observed for the α_1B_ adrenergic receptor^32^ and β_2_ adrenergic receptor^33^, ^34^ among others.^31^ Similar observations to those observed here have been made upon mutation of R3.50 in both the histamine H_4_ ^35^ and cannabinoid CB_2_ receptors.^36^ In both these studies, the mutant receptors retained high‐affinity agonist binding, were unable to couple to G proteins in the presence of agonists and showed no increase in constitutive activity, although effects on β‐arrestin coupling and internalization were not studied.^35^, ^36^ For the bradykinin B_2_ receptor, a R3.50A mutant was also unable to stimulate a second messenger pathways and was not constitutively active.^37^ However, sequestration of the receptor from the membrane, as measured using a radiolabeled agonist, was observed,^37^ suggesting that this may be a more general phenomenon for this mutation.

Through the use of FCS, we demonstrated that A_3_AR R108A could still bind agonists with high affinity. The binding affinity of the fluorescent agonist ABEA‐X‐BY630 was consistent with that observed in previous FCS studies of agonist‐A_3_AR complexes, with similar levels of bound fluorescent agonist and diffusion coefficients.^13^ As observed in many previous FCS studies of fluorescent ligand binding to GPCRs, the autocorrelation curves obtained using ABEA‐X‐BY630 contained three species; a fast diffusing species (*τ_D_*_1_), representing the unbound ligand freely diffusing in solution, and two more slowly diffusing species (*τ_D_*_2_ and *τ_D_*_3_), with the slower diffusing species (*τ_D_*_3_) representing the majority of the total binding.^13^, ^14^ It was originally hypothesized that these two diffusion rates represented two distinct populations of receptor.^13^ Further studies of the A_3_AR, involving the use of an allosteric modulator to increase the relative proportion of *τ_D_*_2_, strongly indicated that *τ_D_*_2_ was generated by the dissociation of the fluorescent ligand from the receptor species during its transit through the confocal volume.^14^ Therefore, the FCS data with fluorescent agonist presented in the current study are only for *τ_D_*_3._

The diffusion coefficient of free and fluorescent agonist‐bound A_3_‐GFP and A_3_ R108A‐GFP were similar (0.13‐0.16 μm^2^/s). This is in line with diffusion coefficients observed previously for the A_3_AR^13^, ^14^ and is slower than expected for an individual receptor freely diffusing in the plasma membrane.^38^ This would indicate that the receptor forms part of a larger complex or has restricted diffusion through interaction with different components of the membrane.^11^ It has previously been shown that pertussis toxin (which inhibits G_i/o_α subunits coupling to receptors) has no effect on the high‐affinity agonist binding at the A_3_AR, indicating that the agonist‐occupied receptors were not coupled to G proteins.^13^ This is supported by the data in the present study, where a receptor that has impaired G protein coupling displayed similar diffusion characteristics to the wild‐type receptor.

Although the A_3_ R108A mutant was unable to effectively couple to G proteins, the combination of confocal, FCS, and BiFC experiments demonstrated that it could recruit β‐arrestin2 and undergo redistribution at the plasma membrane in response to agonist stimulation. Receptor‐arrestin complexes have also been shown to have high affinity for agonists^39^ suggesting that the high affinity of A_3_ R108A for agonists could be due to the formation of a receptor‐arrestin complex, and the allosteric effect of the receptor‐arrestin interaction. Through the use of CRISPR/Cas9 technology, it has recently been shown that, in cells lacking functional Gα proteins, GPCRs can still recruit β‐arrestins and internalize but do not signal to the ERK pathway.^40^ This supports our findings that a receptor that cannot effectively couple to G proteins can still recruit β‐arrestin and internalize. This is further supported by evidence that some GPCRs, including the V_1b_ vasopressin receptor^41^ and follicle‐stimulating hormone receptor,^42^ can recruit β‐arrestin in the absence of phosphorylation by G protein receptor kinases.

Confocal imaging suggests that a large proportion of both A_3_AR and A_3_AR R108A were internalized upon agonist stimulation. This is in contrast to the FCS experiments where an increase in receptor density (N/μm^2^) for both A_3_‐GFP and A_3_ R108A‐GFP was seen upon agonist stimulation, indicating a higher number of individual receptor‐containing complexes at the cell surface. This apparent increase in receptor density is coupled with a slowing in the lateral diffusion of both receptors as demonstrated by the reduction in diffusion coefficient. Taken together, these data suggest that we have sampled areas of the plasma membrane where the diffusion of both wild‐type and mutant A_3_AR is significantly restricted but as there is no concurrent increase in clustering as measured by molecular brightness this may not represent clustering within clathrin‐coated pits prior to internalization. By design, each FCS measurement only detects fluorescent fluctuations in a small circular area of the plasma membrane (~0.1 μm^2^) and requires the fluorescent species to be mobile to detect fluctuations. It is therefore likely that we are selectively monitoring clusters of mobile receptors and the increase in apparent number of fluorescent particles is a consequence of release of receptors from immobilized regions of the membrane upon agonist activation. Molecular brightness data are consistent with this and suggests that there was a modest reorganization of the A_3_ and A_3_ R108A receptors into higher‐order complexes, again suggesting clustering of the receptor.

FCS experiments that selectively monitored the diffusion of specific receptor‐arrestin complexes revealed an additional layer of complexity regarding the diffusion and organization of A_3_AR and A_3_AR R108A. As we are monitoring the diffusion of complemented vYFP, this allowed us to selectively monitor receptor‐arrestin complexes. One drawback of this BiFC technique is that the complementation is essentially irreversible^25^ meaning that the receptor and arrestin will be kept in close proximity even if the interaction is transient. Technologies such as NanoBiT have been developed where the interaction between the two complementing portions of a luciferase protein is low affinity, and therefore, reversible.^43^ However, since luminescence output is low energy it requires very sensitive cameras and long exposures for imaging‐based detection, as a consequence the detection of a luminescence output is not currently achievable using FCS. Under basal conditions, receptor‐arrestin BiFC complexes displayed faster diffusion when compared to receptor‐GFP fusions (A_3_‐GFP, 0.13 ± 0.007 μm^2^/s vs A_3_‐vYc/β‐arrestin2‐vYnL, 0.24 ± 0.03 μm^2^/s). Upon agonist stimulation, both A_3_ and A_3_ R108A‐arrestin complexes slowed compared to basal complexes. This suggests that under basal conditions, receptor‐arrestin complexes (which comprise a small proportion of the total receptor population) are in different compartments compared to the main population of receptor‐GFP fusions and this is unaffected by the introduction of the R108A mutation. In the BiFC FCS experiments, no change in particle number was observed after agonist stimulation although the confocal imaging indicated an increase in BiFC complexes within the cell. As FCS specifically measures complexes at the cell membrane, it is likely that agonist stimulation generates an increased number of receptor‐arrestin complexes that are rapidly internalized. Since there is a significant increase in the molecular brightness of the A_3_‐vYc/β‐arrestin2‐vYnL complexes with NECA treatment, indicating aggregation of these receptor‐arrestin complexes, these data support the findings from FCS experiments that reorganization occurs upon agonist treatment. It has been demonstrated previously that A_3_AR is phosphorylated by GRKs upon agonist stimulation.^44^, ^45^ The molecular brightness data are, therefore, consistent with the possibility that this phosphorylation is required for the movement of receptors into a more confined area prior to internalization. This hypothesis is supported by a recent study which showed, through a combination of FCS and fluorescence recovery after photobleaching, that agonist‐induced reorganization of the μ opioid receptor was mediated by GRK2/3.^12^

In summary, we have demonstrated here that introduction of a R108A mutation into the A_3_AR severely impairs G protein‐dependent signaling by the receptor, yet, preserves its ability to recruit β‐arrestin2 and does not change its movement and organization within the plasma membrane. By comparing the diffusion and membrane organization of wild‐type and R108A A_3_AR under different conditions, we have provided additional support to the theory that the A_3_AR is not pre‐coupled to G proteins at the plasma membrane. In addition, we have provided evidence that prior to internalization, agonist‐stimulated A_3_ARs undergo reorganization within the plasma membrane. This demonstrates the power of combining FCS with population assays to gain insights into the signaling and organization of a GPCR.

## CONFLICT OF INTEREST

The authors declare no conflict of interest.

## AUTHOR CONTRIBUTIONS

L.A Stoddart designed, performed, and analyzed the pharmacology experiments; LE Kilpatrick, R. Corriden, and SJ Briddon designed the FCS experiments; LE Kilpartrick performed and analyzed the FCS experiments; B. Kellam, SJ Briddon, and SJ Hill conceived the study; LA Stoddart, LE Kilpatrick, SJ Briddon, and SJ Hill wrote the manuscript. All authors approved the final version of the manuscript.

## References

[1] Fredholm BB , Ijzerman AP , Jacobson KA , Linden J , Müller CE . International Union of Basic and Clinical Pharmacology. LXXXI. Nomenclature and classification of adenosine receptors—an update. Pharmacol Rev. 2011;63:1‐34. 21303899 10.1124/pr.110.003285 PMC3061413

[2] Borea PA , Varani K , Vincenzi F , et al. The A3 adenosine receptor: history and perspectives. Pharmacol Rev. 2015;67:74‐102. 25387804 10.1124/pr.113.008540

[3] Kazemi MH , Raoofi Mohseni S , Hojjat‐Farsangi M , et al. Adenosine and adenosine receptors in the immunopathogenesis and treatment of cancer. J Cell Physiol. 2018;233:2032‐2057. 28233320 10.1002/jcp.25873

[4] Burnstock G . Purinergic signalling: therapeutic developments. Front Pharmacol. 2017;8:661. 28993732 10.3389/fphar.2017.00661 PMC5622197

[5] Latorraca NR , Venkatakrishnan AJ , Dror RO . GPCR dynamics: structures in motion. Chem Rev. 2017;117:139‐155. 27622975 10.1021/acs.chemrev.6b00177

[6] Manglik A , Kim TH , Masureel M , et al. Structural insights into the dynamic process of beta2‐adrenergic receptor signaling. Cell. 2015;161:1101‐1111. 25981665 10.1016/j.cell.2015.04.043 PMC4441853

[7] Dawaliby R , Trubbia C , Delporte C , et al. Allosteric regulation of G protein‐coupled receptor activity by phospholipids. Nat Chem Biol. 2016;12:35‐39. 26571351 10.1038/nchembio.1960 PMC4718399

[8] DeVree BT , Mahoney JP , Velez‐Ruiz GA , et al. Allosteric coupling from G protein to the agonist‐binding pocket in GPCRs. Nature. 2016;535:182‐186. 27362234 10.1038/nature18324 PMC5702553

[9] Calebiro D , Jobin ML . Hot spots for GPCR signaling: lessons from single‐molecule microscopy. Curr Opin Cell Biol. 2019;57:57‐63. 30522088 10.1016/j.ceb.2018.11.003

[10] Ostrom RS , Insel PA . The evolving role of lipid rafts and caveolae in G protein‐coupled receptor signaling: implications for molecular pharmacology. Br J Pharmacol. 2004;143:235‐245. 15289291 10.1038/sj.bjp.0705930 PMC1575337

[11] Briddon SJ , Kilpatrick LE , Hill SJ . Studying GPCR pharmacology in membrane microdomains: fluorescence correlation spectroscopy comes of age. Trends Pharmacol Sci. 2018;39:158‐174. 29277246 10.1016/j.tips.2017.11.004

[12] Gondin AB , Halls ML , Canals M , Briddon SJ . GRK mediates mu‐opioid receptor plasma membrane reorganization. Front Mol Neurosci. 2019;12:104. 31118885 10.3389/fnmol.2019.00104 PMC6504784

[13] Cordeaux Y , Briddon SJ , Alexander SPH , Kellam B , Hill SJ . Agonist‐occupied A_3_ adenosine receptors exist within heterogeneous complexes in membrane microdomains of individual living cells. FASEB J. 2008;22:850‐860. 17959910 10.1096/fj.07-8180com

[14] Corriden R , Kilpatrick LE , Kellam B , Briddon SJ , Hill SJ . Kinetic analysis of antagonist‐occupied adenosine‐A3 receptors within membrane microdomains of individual cells provides evidence of receptor dimerization and allosterism. FASEB J. 2014;28:4211‐4222. 24970394 10.1096/fj.13-247270 PMC4202110

[15] De Lean A , Stadel JM , Lefkowitz RJ . A ternary complex model explains the agonist‐specific binding properties of the adenylate cyclase‐coupled beta‐adrenergic receptor. J Biol Chem. 1980;255:7108‐7117. 6248546

[16] Samama P , Cotecchia S , Costa T , Lefkowitz RJ . A mutation‐induced activated state of the beta 2‐adrenergic receptor. Extending the ternary complex model. J Biol Chem. 1993;268:4625‐4636. 8095262

[17] Tao YX . Constitutive activation of G protein‐coupled receptors and diseases: insights into mechanisms of activation and therapeutics. Pharmacol Ther. 2008;120:129‐148. 18768149 10.1016/j.pharmthera.2008.07.005 PMC2668812

[18] Parnot C , Miserey‐Lenkei S , Bardin S , Corvol P , Clauser E . Lessons from constitutively active mutants of G protein‐coupled receptors. Trends Endocrinol Metab. 2002;13:336‐343. 12217490 10.1016/s1043-2760(02)00628-8

[19] Ballesteros JA , Weinstein H , Stuart CS . [19] Integrated methods for the construction of three‐dimensional models and computational probing of structure‐function relations in G protein‐coupled receptors. In: PD Conn , SC Sealfon (Eds.), Methods in Neurosciences. Vol. 25. Cambridge, USA: Academic Press; 1995:366‐428.

[20] Chen A , Gao ZG , Barak D , Liang BT , Jacobson KA . Constitutive activation of A_3_ adenosine receptors by site‐directed mutagenesis. Biochem Biophys Res Commun. 2001;284:596‐601. 11396942 10.1006/bbrc.2001.5027 PMC3626079

[21] Stoddart LA , Kellam B , Briddon SJ , Hill SJ . Effect of a toggle switch mutation in TM6 of the human adenosine A(3) receptor on Gi protein‐dependent signalling and Gi‐independent receptor internalization. Br J Pharmacol. 2014;171:3827‐3844. 24750014 10.1111/bph.12739 PMC4128046

[22] Donaldson J , Brown AM , Hill SJ . Influence of rolipram on the cyclic 3’,5’‐adenosine‐monophosphate response to histamine and adenosine in slices of guinea‐pig cerebral‐cortex. Biochem Pharmacol. 1988;37:715‐723. 2829922 10.1016/0006-2952(88)90146-3

[23] Kilpatrick LE , Briddon SJ , Hill SJ , Holliday ND . Quantitative analysis of neuropeptide Y receptor association with beta‐arrestin2 measured by bimolecular fluorescence complementation. Br J Pharmacol. 2010;160:892‐906. 20438572 10.1111/j.1476-5381.2010.00676.x PMC2901518

[24] Middleton RJ , Briddon SJ , Cordeaux Y , et al. New fluorescent adenosine A1‐receptor agonists that allow quantification of ligand−receptor interactions in microdomains of single living cells. J Med Chem. 2007;50:782‐793. 17249651 10.1021/jm061279i

[25] Rose RH , Briddon SJ , Holliday ND . Bimolecular fluorescence complementation: lighting up seven transmembrane domain receptor signalling networks. Br J Pharmacol. 2010;159:738‐750. 20015298 10.1111/j.1476-5381.2009.00480.x PMC2829200

[26] Sungkaworn T , Jobin ML , Burnecki K , Weron A , Lohse MJ , Calebiro D . Single‐molecule imaging reveals receptor‐G protein interactions at cell surface hot spots. Nature. 2017;550:543‐547. 29045395 10.1038/nature24264

[27] Yanagawa M , Hiroshima M , Togashi Y , et al. Single‐molecule diffusion‐based estimation of ligand effects on G protein‐coupled receptors. Sci Signal. 2018;11:eaoo1917. 10.1126/scisignal.aao1917 30228224

[28] Isberg V , de Graaf C , Bortolato A , et al. Generic GPCR residue numbers—aligning topology maps while minding the gaps. Trends Pharmacol Sci. 2015;36:22‐31. 25541108 10.1016/j.tips.2014.11.001 PMC4408928

[29] Palczewski K , Kumasaka T , Hori T , et al. Crystal structure of rhodopsin: a G protein‐coupled receptor. Science. 2000;289:739‐745. 10926528 10.1126/science.289.5480.739

[30] Carpenter B , Nehmé R , Warne T , Leslie AG , Tate CG . Structure of the adenosine A_2A_ receptor bound to an engineered G protein. Nature. 2016;536:104‐107. 27462812 10.1038/nature18966 PMC4979997

[31] Rovati GE , Capra V , Neubig RR . The highly conserved DRY motif of class A G protein‐coupled receptors: beyond the ground state. Mol Pharmacol. 2007;71:959‐964. 17192495 10.1124/mol.106.029470

[32] Scheer A , Fanelli F , Costa T , De Benedetti PG , Cotecchia S . The activation process of the alpha1B‐adrenergic receptor: potential role of protonation and hydrophobicity of a highly conserved aspartate. Proc Natl Acad Sci USA. 1997;94:808‐813. 9023338 10.1073/pnas.94.3.808 PMC19595

[33] Valentin‐Hansen L , Groenen M , Nygaard R , Frimurer TM , Holliday ND , Schwartz TW . The arginine of the DRY motif in transmembrane segment III functions as a balancing micro‐switch in the activation of the β2‐adrenergic receptor. J Biol Chem. 2012;287:31973‐31982. 22843684 10.1074/jbc.M112.348565 PMC3442529

[34] Ballesteros JA , Jensen AD , Liapakis G , et al. Activation of the beta 2‐adrenergic receptor involves disruption of an ionic lock between the cytoplasmic ends of transmembrane segments 3 and 6. J Biol Chem. 2001;276:29171‐29177. 11375997 10.1074/jbc.M103747200

[35] Schneider EH , Schnell D , Strasser A , Dove S , Seifert R . Impact of the DRY motif and the missing "ionic lock" on constitutive activity and G‐protein coupling of the human histamine H4 receptor. J Pharmacol Exp Ther. 2010;333:382‐392. 20106995 10.1124/jpet.109.163220

[36] Feng W , Song ZH . Effects of D3.49A, R3.50A, and A6.34E mutations on ligand binding and activation of the cannabinoid‐2 (CB2) receptor. Biochem Pharmacol. 2003;65:1077‐1085. 12663043 10.1016/s0006-2952(03)00005-4

[37] Leschner J , Wennerberg G , Feierler J , et al. Interruption of the ionic lock in the bradykinin B2 receptor results in constitutive internalization and turns several antagonists into strong agonists. J Pharmacol Exp Ther. 2013;344:85‐95. 23086229 10.1124/jpet.112.199190

[38] Ganguly S , Chattopadhyay A . Cholesterol depletion mimics the effect of cytoskeletal destabilization on membrane dynamics of the serotonin1A receptor: a zFCS study. Biophys J. 2010;99:1397‐1407. 20816051 10.1016/j.bpj.2010.06.031 PMC2931730

[39] Gurevich VV , Pals‐Rylaarsdam R , Benovic JL , Hosey MM , Onorato JJ . Agonist‐receptor‐arrestin, an alternative ternary complex with high agonist affinity. J Biol Chem. 1997;272:28849‐28852. 9360951 10.1074/jbc.272.46.28849

[40] Grundmann M , Merten N , Malfacini D , et al. Lack of beta‐arrestin signaling in the absence of active G proteins. Nat Commun. 2018;9:341. 29362459 10.1038/s41467-017-02661-3 PMC5780443

[41] Perkovska S , Méjean C , Ayoub MA , et al. V(1b) vasopressin receptor trafficking and signaling: role of arrestins, G proteins and Src kinase. Traffic. 2018;19:58‐82. 29044966 10.1111/tra.12535

[42] Kara E , Crépieux P , Gauthier C , et al. A phosphorylation cluster of five serine and threonine residues in the C‐terminus of the follicle‐stimulating hormone receptor is important for desensitization but not for beta‐arrestin‐mediated ERK activation. Mol Endocrinol. 2006;20:3014‐3026. 16887887 10.1210/me.2006-0098

[43] Dixon AS , Schwinn MK , Hall MP , et al. NanoLuc complementation reporter optimized for accurate measurement of protein interactions in cells. ACS Chem Biol. 2016;11:400‐408. 26569370 10.1021/acschembio.5b00753

[44] Ferguson G , Watterson KR , Palmer TM . Subtype‐specific regulation of receptor internalization and recycling by the carboxyl‐terminal domains of the human A_1_ and rat A_3_ adenosine receptors: Consequences for agonist‐stimulated translocation of arrestin3. Biochemistry. 2002;41:14748‐14761. 12475223 10.1021/bi0262911

[45] Palmer TM , Benovic JL , Stiles GL . Agonist‐dependent phosphorylation and desensitization of the rat A_3_ adenosine receptor—evidence for a G protein‐coupled receptor kinase‐mediated mechanism. J Biol Chem. 1995;270:29607‐29613. 7494005 10.1074/jbc.270.49.29607

